# The Proprotein Convertase Furin Contributes to Rhabdomyosarcoma Malignancy by Promoting Vascularization, Migration and Invasion

**DOI:** 10.1371/journal.pone.0161396

**Published:** 2016-08-22

**Authors:** Patricia Jaaks, Valentina D’Alessandro, Nicole Grob, Sina Büel, Katarina Hajdin, Beat W. Schäfer, Michele Bernasconi

**Affiliations:** 1 Department of Oncology, University Children’s Hospital Zurich, Zurich, Switzerland; 2 Children’s Research Center, University Children’s Hospital Zurich, Zurich, Switzerland; Ospedale Pediatrico Bambino Gesu, ITALY

## Abstract

The proprotein convertase (PC) furin cleaves precursor proteins, an important step in the activation of many cancer-associated proteins. Substrates of furin and furin-like PCs play a role in proliferation, metastasis and invasion. Some of them are involved in the progression of the pediatric soft tissue sarcoma rhabdomyosarcoma (RMS). In this study, we show that PCs, and in particular furin, are expressed in RMS cell lines. To investigate the functional role of furin, we generated RMS cell lines with modulated furin activity. Silencing or stable inhibition of furin delayed tumor growth in Rh30 and RD xenografts *in vivo*, and was correlated with lower microvessel density. Reduced furin activity also decreased migration and invasion abilities *in vitro*, and inhibition of furin in RMS cells diminished processing of IGF1R, VEGF-C, PDGF-B and MT1-MMP, leading to lower levels of mature proteins. Furthermore, we found that furin activity is required for proper IGF signaling in RMS cells, as furin silencing resulted in reduced phosphorylation of Akt upon IGF1 stimulation. Taken together, our results suggest that furin plays an important role in the malignant phenotype of RMS cells by activating proteins involved in tumor growth and vascularization, metastasis and invasion.

## Introduction

Proproteins are synthesized as inactive precursor proteins and require limited proteolysis to be converted into bioactive proteins and peptides. They are usually cleaved at the general consensus motif R/K-X_n_-R/K↓, where n = 0, 2, 4 or 6 and X rarely Cys or Pro [1]. Seven mammalian proprotein convertases (PCs) cleaving substrates at dibasic motifs have been described: PC1/3, PC2, furin, PC4, PC5, PACE4 and PC7. In the secretory pathway, PCs mediate the tissue-specific endoproteolytic activation of precursor proteins, including hormones, neuropeptides, growth factors and their receptors, adhesion molecules, bacterial toxins and viral glycoproteins [2]. Consequently, deregulated activity of PCs has been associated with pathological conditions like Alzheimer’s disease [3], endocrinopathies [4] or cancer [5,6]. Increased PC activity correlates with higher aggressiveness of diverse cancer types like prostate cancer, colon carcinoma or small cell lung carcinoma [7,8,9].

Furin was the first described PC and is active within the constitutive secretory pathway [10]. This calcium-dependent protease cleaves different proteins implicated in cancer progression and metastasis such as the insulin-like growth factors receptor 1 (IGF1R) [8], the growth factors vascular endothelial growth factor C (VEGF-C) and platelet derived growth factor (PDGF) [11] and membrane-type 1 matrix metalloprotease 1 (MT1-MMP) [12,13]. Furthermore, elevated levels of furin are associated with head and neck cancer, breast, lung and colon cancers [14,15,16,17].

In a previous study, we identified cell surface furin as potential receptor for peptides that specifically homed to rhabdomyosarcoma (RMS) cells *in vitro* and *in vivo* [18]. RMS represents with around 55% of all cases the most common soft tissue sarcoma in children and adolescents [19]. Based on histology, pediatric RMS are grouped into two main subtypes: embryonal RMS (eRMS) and alveolar RMS (aRMS). eRMS occurs in around 60%-70% of all patients and is associated with a rather good prognosis, whereas 20% of patients present with aRMS, having a 5-year overall survival of only 20%-30% [20]. Prognosis for patients with metastasis at time of diagnosis is particularly poor with a 5-year overall survival rate of less than 12% [20]. Complementing or improved treatment strategies are urgently needed and thus many recent efforts have been concentrated on the identification of key pathways that drive RMS progression. In 80% of aRMS tumor formation is driven by expression of the chimeric transcription factor PAX3/7-FOXO1 [21,22,23], which induces expression of a specific gene expression signature [24,25,26]. Many receptor tyrosine kinases are direct targets of PAX3/7-FOXO1, including IGF1R, VEGFR and PDGFR [27,28] and RMS progression is characterized by aberrant activation of growth factor signaling pathways [29]. As furin is involved in the maturation and activation of many components of these pathways, we hypothesized that furin activity is important for the malignant phenotype of RMS cells. Therefore, we assessed the expression levels of furin and other PCs in pediatric sarcoma cell lines and generated RMS cell lines with different levels of furin activity. We subsequently used these cell lines to examine tumor growth *in vivo* and to assess processing of furin substrates, migration, and invasion *in vitro*. Our investigations showed that furin promotes tumor growth as well as migration and invasion abilities in RMS by cleaving key proteins involved in RMS cell growth, tumor vascularization, cell motility and invasiveness. Therefore, we propose the PC furin as new therapeutic target for treatment of RMS.

## Materials and Methods

### Ethics Statement

All animal experiments were approved and monitored by the veterinary office of the Canton of Zurich according to the Swiss Federal Law (permits 158/2009; 208/2011). Mice were euthanized according to the approved protocol, either by terminal anesthesia with pentobarbital and intracardial perfusion, or by controlled CO_2_ asphyxia.

### Statistical analysis

Statistical analysis was performed using GraphPad Prism. Data are expressed as mean ± standard deviation (SD). Statistical significance was tested with unpaired two-tailed Student’s t-tests, or for multiple comparison analysis of variance (ANOVA). The differences were considered to be significant if p<0.05.

### Cell lines and cell culture

Osteosarcoma cell lines SAOS, LM5, HOS, 143B, MG63, M8, HU09 and M132 were kindly provided by Roman Muff (University Hospital Balgrist, Zurich, Switzerland). Ewing sarcoma cell lines TC71, A673, RD-ES, SK-ES and SKNMC, as well as the RMS cell line RUCH-2, were kindly provided by Beat Schäfer (University Children’s Hospital Zurich, Zurich, Switzerland). Other RMS cell lines were kindly provided by following sources: Rh3 and Rh5 by Susan Ragsdale (St. Jude Children’s Research Hospital, Memphis, TN, USA); RD, Rh36, Rh18, Rh4 and Rh41 by Peter Houghton (The Research Institute at Nationwide Children’s Hospital, Columbus, OH, USA); TTC442 and Birch by Timothy Triche (Children’s Hospital Los Angeles, Los Angeles, CA, USA); NRS-1 (RIKEN Cell Bank); RMS and SCMS-RMZ by Janet Shipley (The Institute of Cancer Research, London, UK); Rh28 by Corinne Linardic (Duke University School of Medicine, Durham, NC, USA); RMS13 by Roland Kappler (LMU, Munich, Germany); CW9019 by Soledad Gallego (Hospital Universitari Vall d’Hebron, Barcelona, Spain); KFR by Jindrich Cinatl (Frankfurter Stiftung für krebskranke Kinder, Frankfurt, Germany); RC2 by Pier-Luigi Lollini (University of Bologna, Bologna, Italy) and RhJT by Scott Diede (Fred Hutchinson Cancer Research Center, Seattle, WA, USA). Rh30 cells and furin activity deficient LoVo cells (colorectal adenocarcinoma) were purchased from ATCC (LGC Promochem, Molsheim Cedex, France). All cells were maintained under proliferating conditions in high glucose DMEM medium (Sigma-Aldrich, Buchs, Switzerland) supplemented with 10% fetal calf serum (Bioconcept, Switzerland) in 5% CO_2_ at 37°C.

### Plasmids and Transfections

pcDNA3.1(+) vectors encoding the full length furin (fur) [30] or α1-AT Portland (pdx), were generous gifts of Andres JP Klein-Szanto (Fox Chase Cancer Center, Philadelphia, PA). For stable transfections, 2.5-5x10^5^ RD, Rh4 or Rh30 cells were transfected with 2 μg DNA according to the jetPRIME™ short protocol (Polyplus Transfection, Illkirch, France). Selection with 1 mg/ml G418 (Promega, Wallisellen, Switzerland) was started 48h post transfection and was continued for 14 days. RMS cells transfected with empty pcDNA3.1(+) vector were used as control in qRT-PCR experiments.

### Transient and stable silencing of furin

To transiently silence furin, 10 nM siRNA (Silencer Select, Ambion, Austin, Texas) were reverse transfected into RD cells using the transfection reagent INTERFERin (Polyplus Transfection, Illkirch, France) according to manufacturer’s instructions. Following siRNAs were used: scrambled (scr, ID: AM4637), siFurin1 and siFurin2 (siFur1, ID: s9987; siFur2, ID: s9988). For stable silencing of furin in RD, Rh4, and Rh30 cells, mission shRNA lentiviral particles (Sigma-Aldrich) based on a pLKO.1 plasmid backbone were used. Lentiviral particles of five clones (SHCLNV-NM 002569 Human, TRCN0000075238-42) targeting different sequences of furin RNA or lentiviral particles non-target shRNA (SHC002V) were added at an MOI of 5 to RMS cells for 24h. Selection was started on day 5 with 2 μg/ml of puromycin (Invitrogen, Basel, Switzerland). Non-target shRNA RMS cells were used as control in qRT-PCR experiments.

### Animal models

To study *in vivo* growth, 5x10^6^ RMS cells in 150 μl of PBS were injected s.c. into NOD/Scid IL2rg^-/-^ mice (Charles River) at 5–6 weeks of age, under anesthesia induced by intraperitoneal injection of 100 mg/kg ketamine (Ketalar, Parke-Davis, Morris Plains, NJ) and 16 mg/kg xylazine (Rompun, Bayer HealthCare, Leverkusen, Germany). For measurement of the tumor size, both diameters (d) of the ellipsoidal tumors were measured with a caliper and the tumor volume was calculated using the formula V = (4/3)πr^3^, whereby r = ((d_1_+d_2_)/4). Mice were sacrificed when a tumor size of 1000 mm^3^ was reached. Mice were perfused with PBS after terminal anesthesia with 420 mg/kg pentobarbital (Esconarkon; Streuli Pharma, Uznach, Switzerland). Tumors were dissected, fixed for 24 hours in 4% PFA (Thermo Scientific, Switzerland) and embedded in paraffin, or stabilized in 30% sucrose (Sigma-Aldrich, Basel, Switzerland) and frozen in O.C.T embedding medium (Leica Microsystems, Heerbrugg, Switzerland), as indicated.

### Immunofluorescence

Immunofluorescence to detect angiogenic blood vessels in RMS xenograft tumors was performed on 5 μm fresh frozen section from O.C.T. embedded samples. Sections were washed in TBS/0.2% Tween-20 for 15 min and stained with CD31 antibody (550274, BD Pharmingen, Allschwil, Switzerland) diluted 1:100 in antibody diluent solution (Zytomed Systems GmbH, LabForce, Nunningen, Switzerland). Secondary Alexa Fluor 594-labelled goat anti-rat IgG was used for detection (A-11007, Invitrogen). Sections were washed twice with PBS and mounted with Vectashield Mounting medium containing DAPI (Reactolab SA, Servion, Switzerland).

### Immunohistochemistry

Immunohistochemistry of PFA-fixed tumors was performed by Sophistolab (Muttenz, Switzerland) on an automated Leica BondMax system using Bond Polymer Refine Detection (DS9800, Leica Microsystems, Newcastle, UK) including all buffer-solutions from Leica processed according to the manufacturer’s instructions. Paraffin slides were dewaxed, pretreated with ER-Solution 2 and incubated with the following antibodies: polyclonal rabbit anti-furin (ab28547, Abcam, Cambridge, UK) used at a dilution of 1:3000 after pretreatment for 10 min at 95°C; anti-CD31 (ab28364, Abcam) used at a dilution of 1:100 after antigen pretreatment for 20 min at 100°C.

### Quantitative RT-PCR

Total RNA was extracted from RMS cells or tumor tissue using the RNeasy Kit (Qiagen, Hombrechtikon, Switzerland) including a DNase treatment step. 1 μg total RNA was reverse-transcribed with random primers using the Omniscript Reverse Transcription Kit (Qiagen). qRT-PCR detection of furin, α_1_-PDX and the house keeping gene GAPDH was performed with assay-on-demand Hs00965485_g1, Hs01097800_m1 or Hs99999905_m1, respectively (Applied Biosystems, Basel, Switzerland), and normalized to GAPDH.

### Immunoblot

RMS cells were lysed in denaturing buffer (50 mM TrisHCl, 0.1% Triton X-100, 1 mM EDTA, 1 mM EGTA, 50 mM NaF, 5 mM Na_4_P_2_O_7_, 10 mM C_3_H_7_Na_2_O_6_P, 1 mM sodium orthovanadate) supplemented with 1 mM PMSF (phenylmethylsulfonyl fluoride) and Roche Complete Protease inhibitor (Roche, Rotkreuz, Switzerland). Cell lysates were centrifuged for 10 min at 10’000 rpm, the supernatant was collected and protein concentrations were determined in a Bradford assay (Bio-Rad laboratories, Reinach, Switzerland). Total cell extracts were separated on 4–12% NuPAGE Bis-Tris gels (Invitrogen) and blotted on nitrocellulose membranes (0.1 μm; Schleicher & Schuell, Dassel, Germany). Blots were blocked with 5% milk in TBS/0.05% Tween-20, incubated with the first antibody overnight at 4°C, and with the corresponding HRP-conjugated secondary antibody for 1h at RT. Enhanced chemiluminescence detection system SuperSignal West Femto (Pierce, Perbio Science, Lausanne, Switzerland) was used for signal detection. The following antibodies were used: anti-furin mouse monoclonal MON-152 (ALX-803-017-R100, 1:750, Alexis Corporation, Lausen, Switzerland), anti-VEGF-C rabbit polyclonal (sc-25783, 1:200; Santa Cruz, LabForce, Switzerland), anti-IGF1Rβ rabbit polyclonal (9750S, 1:1000, Cell Signaling, Bioconcept), anti-MT1-MMP rabbit polyclonal (NG1726963, 1:750, Millipore), anti-PDGF goat polyclonal (06–127, 1:500, Millipore), anti-pAkt polyclonal rabbit (9271S, 1:1000, Cell Signaling, Bioconcept) and anti-Akt polyclonal rabbit (9272, 1:1000, Cell Signaling, Bioconcept). Mouse anti-β-actin (A5316, 1:5000, Sigma-Aldrich) or anti-α-tubulin (T4026, 1:1000, Sigma-Aldrich) were used for loading controls.

### Furin activity test

To measure the activity of furin, an immobilized furin assay was performed as described previously [31]. 10^6^ RMS cells were treated with 5x lysis/ reaction buffer (500 mM HEPES pH 7.0, 2.5% Triton X-100, 5 mM calcium chloride, 5 mM β-mercaptoethanol), incubated on ice for 10 min and centrifuged. Black FluoroNunc 96 well plates (MaxiSorp surface, Nunc, Thermo Scientific) were coated with goat anti human furin antibody (AF1503, R&D Systems) at 10 μg/ml in 50 mM Na_2_CO_3_, pH 9.6 (50 μl/ well) for 8h at RT, protected from light. After blocking for 1h at RT (0.5% sucrose, 0.5% Tween-20 in PBS), cell lysates were plated in triplicates and incubated over night at 4°C. Wells were washed, 180 μL 1.1x lysis buffer per well added, and the reaction was started by adding 20 μl/well of 1 mM furin fluorogenic Boc-RVRR-AMC (ALX-260-040 Alexis, Enzo Life Sciences). Fluorescence (ex: 380 nm; em: 460 nm) was measured using a Bio-TEK Multi-Mode Microplate Reader (Witec AG, Luzern, Switzerland).

### Cell migration

RMS cells were cultured to 90% confluency in 6-well plates and three separate wounds per well were introduced with a 200 μl pipette tip. Six pictures per well were taken at 10x magnification at different time points (0, 4, 16, 24 hours), analyzed with the program TScratch [32], and the percentage of closed wound was calculated over the time point 0h.

### Invadopodia assay

Invadopodia assays were performed following the manufacturer’s instructions (ECM670, Millipore, Zug, Switzerland). For the experiment, 1.2x10^3^ cells were seeded onto FITC-labeled gelatin into 8-chamber slides and incubated at 37°C for 24h or 48h. Cells were fixed with 4% formaldehyde for 30 min, washed and stained with a solution of TRITC-phalloidin (2 μg/ml) and DAPI (1 μg/ml) in fluorescent staining buffer (PBS with 2% blocking serum and 0.25% Triton X-100) for 1h at RT, protected from light. Coverslips were mounted on the slides using hard set mounting medium (Reactolab) and pictures were taken at 20x magnification with a fluorescence microscope (Axioskop 2 Mot, Zeiss, Feldbach, Switzerland). Image analysis was performed using the program ImageJ [33].

### IGF1 stimulation

RD cells were transiently transfected with anti-furin siRNA (siFur1 and siFur2) or control (scr) siRNA, as described above. After 24h of starvation (DMEM, 0.2% FBS), cells were stimulated for 10 min with 50 ng/mL IGF1 (ab73455, Abcam). Cells were washed with ice-cold PBS and snap frozen in liquid nitrogen. Whole cell lysates were prepared as described above and IGF1Rβ, Akt and phosphorylated Akt were analyzed by immunoblotting.

## Results

### Furin is expressed and active in RMS cell lines

**Fig 1:**
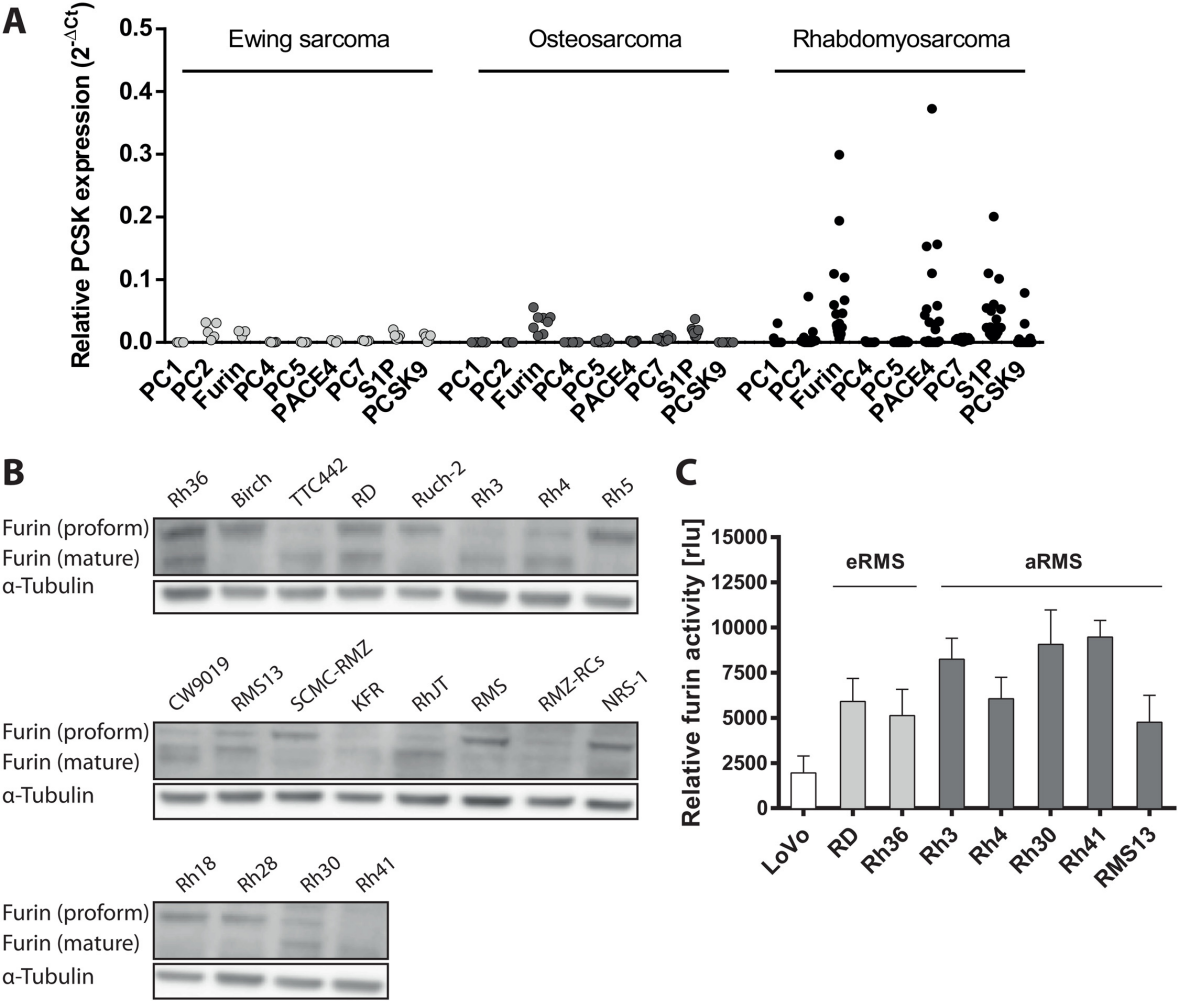
Furin is highly expressed in rhabdomyosarcoma (RMS) cell lines. A) mRNA levels of all nine proprotein convertases (PCs) were determined by qRT-PCR in 5 Ewing sarcoma, 8 osteosarcoma and 20 rhabdomyosarcoma (RMS) cell lines. Shown are levels relative to GAPDH expression. B) Protein levels of proform and mature furin were assessed by immunoblotting in 20 different RMS cell lines. C) Endogenous furin activity of selected RMS cell lines: Rh36, RD (eRMS), Rh3, Rh4, Rh30, Rh41 and RMS13 (aRMS). Furin activity deficient cells LoVo cells serve as negative control. Furin was captured from cell lysates on anti-furin antibody coated plates and furin activity was measured by addition of the fluorogenic substrate Boc-RVRR-AMC after 6h. Displayed are values normalized by background subtraction.

To assess the presence of furin and other PCs in pediatric sarcomas we examined the mRNA levels of all nine known PCs in 5 Ewing sarcoma, 8 osteosarcoma and 20 rhabdomyosarcoma (RMS) cell lines (list of cell lines and relative values in S1 Table). Overall, RMS cell lines showed higher levels of PCs than Ewing sarcoma or osteosarcoma cell lines. Particularly furin, PACE4 and S1P were highly expressed, with furin being the PC with the most consistently elevated mRNA level (Fig 1A and S2 Fig). No consistent pattern of expression could be observed between furin and PACE4 in the different cell lines, and no clear difference between alveolar RMS and embryonal RMS cell lines was evident. Subsequently, we analyzed maturation status of furin protein in all RMS cell lines and found detectable amounts of mature furin in approximately half of the cell lines tested (Fig 1B). From those, we chose seven cell lines (two eRMS, five aRMS) and measured activity of furin based on an immunocapture fluorogenic substrate assay. These experiments showed that *in vitro* furin activity could be detected in all cell lines showing a detectable band for mature furin (Fig 1C). Absolute activity levels, did not completely correlate with total furin protein expression as detected by immunoblot, possibly because of differences in protein extraction efficiency or stability between immunoblot and activity assay. It is interesting to note, however, that activity levels, seem to correlate with the ratio between mature furin and proform rather than with levels of mature form alone.

### Establishment and validation of RMS cells with modulated furin activity

**Fig 2:**
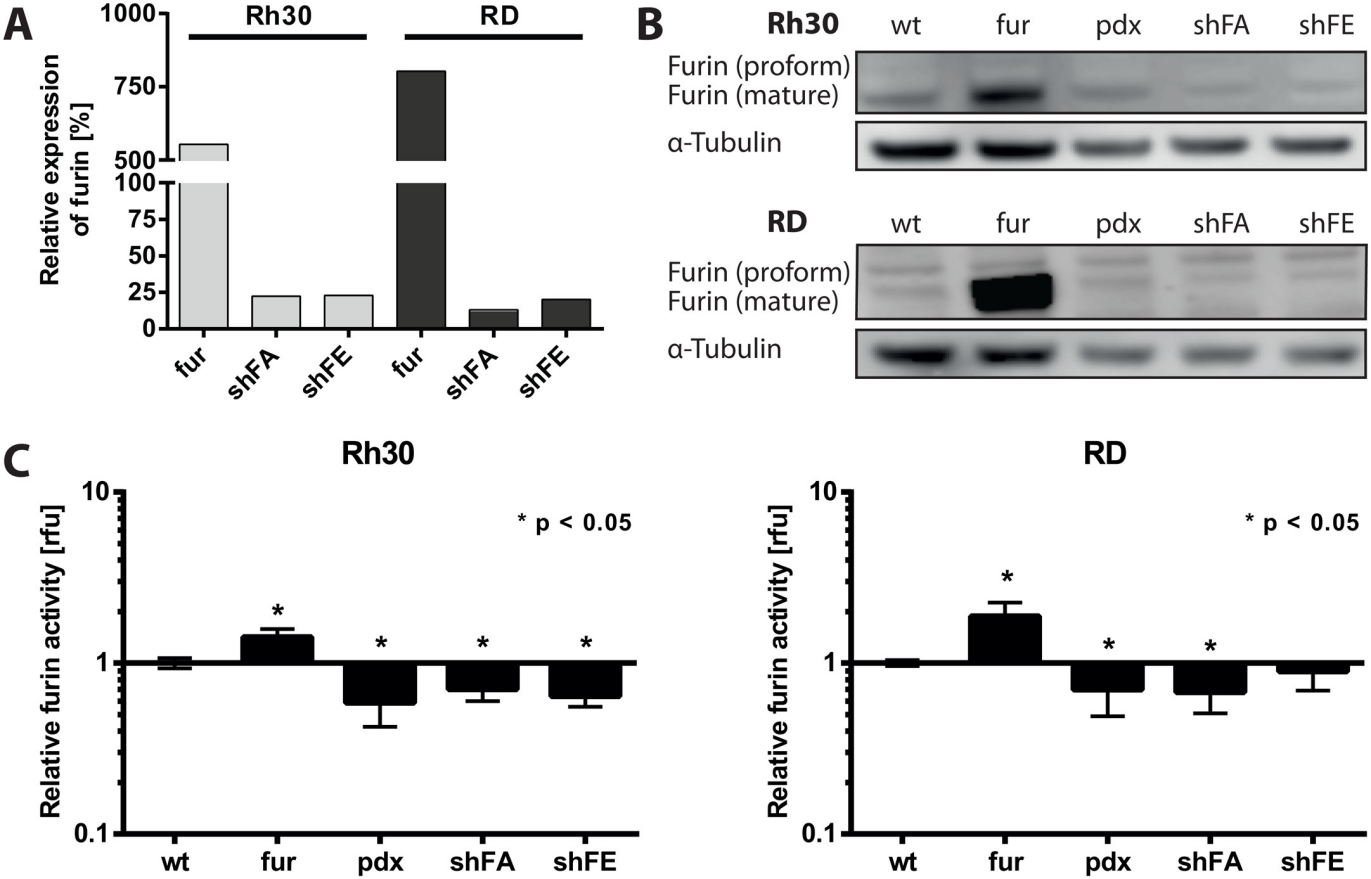
Generation and validation of RMS cells with stable modulation of furin. Rh30 and RD cells were stably transfected with pcDNA3.1(+) full length furin (fur) or α1-AT Portland (pdx). shRNA lentiviral particles were employed for stable furin silencing (shFA, shFE). A) Furin mRNA levels were quantified by qRT-PCR. Expression levels were normalized to GAPDH and to furin expression of the respective control cells (empty vector or scrambled shRNA). B) Levels of furin protein in stable Rh30 and RD cell lines were assessed by immunoblotting with antibody MON-152. C) Assessment of furin activity in furin overexpressing or silenced cell lines. The results were normalized over the respective wt cells on a log_10_ scale. Data represent three independent experiments. Depicted are mean values ± SD. Two-way ANOVA, p<0.05.

To investigate the function of furin in RMS, we selected two cell lines, one for each RMS subtype, Rh30 (aRMS) and RD (eRMS), and generated stable cell lines with either increased furin activity by overexpression of furin (fur), or with decreased furin activity by overexpression of the pan-PC inhibitor α1-antitrypsin Portland variant (pdx). In addition, to achieve a specific inhibition of furin, we downregulated furin expression with two distinct shRNAs targeting furin (shFA and shFE). Furin and pdx overexpression, as well as furin silencing by shRNA, were first validated at the mRNA level by qRT-PCR. As expected, RMS cells overexpressing furin (RMS fur) showed an increased level of furin expression compared to the respective empty vector cells (Fig 2A; Rh30 fur: 5.5-fold, RD fur: 8-fold). *De novo* expression of pdx was confirmed by qRT-PCR (data not shown). Both furin shRNAs, shFA and shFE, on the other hand, decreased furin expression around 5-fold over control (scr) shRNA (Fig 2A). Next, furin protein levels were analyzed by immunoblotting, confirming overexpression of furin in Rh30 fur and RD fur, wild type levels in Rh30 pdx and RD pdx. The decrease of furin expression was not so evident, despite a clear downregulation at the mRNA level (Fig 2B). Therefore, to validate the silencing approach, and to verify the correlation between the presence of furin protein and its activity, we performed a whole cell lysate specific furin activity assay [31]. Furin overexpression led to a significant increase in furin activity (Fig 2C; Rh30 fur: 1.8-fold, RD fur: 2.5-fold). Inhibition of furin through pdx expression, or reduction of furin expression by shRNA, both efficiently reduced furin activity (Fig 2C). In order to exclude possible compensation of lowered furin activity through increased expression of related PCs, we analyzed mRNA levels of all PC family members and did not observe any increase of other PCs upon loss of functional furin (data not shown). Thus, successful modulation of furin activity in aRMS and eRMS cell lines Rh30 and RD could be confirmed at mRNA, protein and, most importantly, activity level allowing the use of these cell lines for further investigations on the role of furin in RMS progression.

### Furin activity correlates with tumor growth and microvessel formation *in vivo*

**Fig 3:**
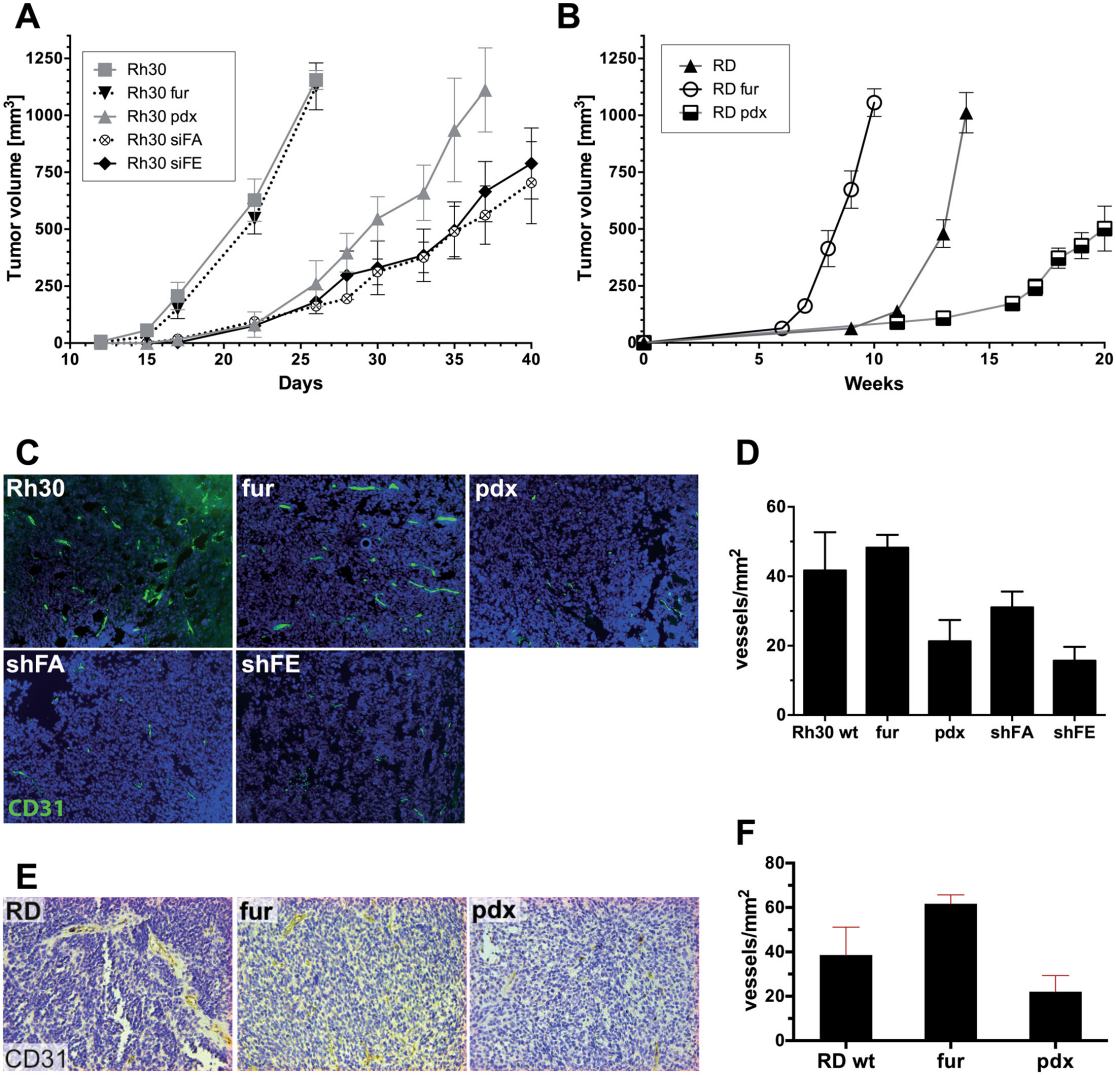
Furin activity positively correlates with tumor growth and microvessel density. 5 million RMS cells with stable modulation of furin activity were injected s.c. into NOD/Scid IL2reg^-/-^ and tumor growth was monitored over time. A) Tumor growth in aRMS Rh30 xenografts. Tumors took on average 20–35 days to reach a size of 500 mm^3^. Each group consisted of 6 mice. B) Tumor growth in eRMS RD xenografts. Tumors took on average 7–19 weeks to reach a size of 500 mm^3^. Each group consisted of 10 mice. C) Histological analysis of microvessel density in Rh30 xenografts. Tumors were collected when they reached a size of 200–400 mm^3^ and sections were stained for CD31 by immunofluorescence. Blue: Nuclear DAPI staining. Representative pictures are shown (magnification: 10x). D) Microvessel density in Rh30 tumors was assessed as vessels per mm^2^. The graph shows the quantification averaged per picture. Depicted are mean values ± SD. E) Histological analysis of microvessel density of RD tumors. Tumors were collected at the end point and paraffin section were stained for furin and with H&E. Shown are representative pictures (magnification: 10x). F) Microvessel density in RD tumors was assessed as vessels per mm^2^. Three pictures per section, total one section. The graph shows the quantification averaged per picture. Depicted are mean values ± SD.

Having successfully established Rh30 and RD cells with different levels of furin activity, we used these cell lines to investigate the impact of furin on RMS tumor growth *in vivo*. RMS tumors were generated from the different RMS cell lines by subcutaneous engraftment in NOD/Scid IL2rg^-/-^ mice, and tumor growth was monitored. Wild type aRMS Rh30 cells grew faster than wild type eRMS RD cells; Rh30 tumors were followed over a period of 40 days, while RD tumors were followed over a period of 20 weeks. No difference in tumor growth was observed between Rh30 wt and Rh30 fur, whereas decreased furin activity resulted in a clear delay in tumor growth (Fig 3A); Rh30 pdx, Rh30 shFA and Rh30 shFE tumors took approximately 1.5-times longer to reach a size of 500 mm^3^ compared to Rh30 wt (Fig 3A). The time period to reach 500 mm^3^ was 22 days for Rh30 wt and Rh30 fur, 30 days for Rh30 pdx, and 35 days for Rh30 shFA and Rh30 shFE. The same trend was observed for RD tumors, whereby RD tumors with decreased furin activity grew slower than RD wt tumors and took almost 30% longer to reach a size of 500 mm^3^ (Fig 3B; RD wt: 14 weeks, RD pdx: 20 weeks). Remarkably, in RD xenografts the increase in furin expression led to faster tumor growth; RD fur tumors reached a size of 500 mm^3^ in 8 weeks compared to 14 weeks required by RD wt to reach the same size, which corresponds to a 40% decrease (Fig 3B). This acceleration in tumor growth upon furin overexpression was not observed in the alveolar RMS Rh30 tumor model. Assessment of furin protein levels by immunoblotting revealed that expression of furin in Rh30 fur *in vivo* was only moderately increased as compared to tumors derived from Rh30 wt cell lines (S2 Fig), contrary to what was observed *in vitro* (Fig 2B). This might be due to an intrinsic increase of furin protein levels in Rh30 tumors grown *in vivo*. Importantly, immunoblots showed that the silencing of furin in shFA and shFE was maintained *in vivo* in Rh30 cells derived tumors (S2 Fig). Taken together, these results support the hypothesis that furin activity contributes to RMS growth *in vivo*. Moreover, furin silencing and inhibition led to delayed tumor onset rather than to a complete tumor growth inhibition, hinting at a role of furin in early steps of tumor growth *in vivo*.

Since we observed a delayed initiation of tumor growth upon reduction of furin activity, we were interested to elucidate the involved molecular mechanisms. Therefore, we analyzed the microvessel density (MVD) on sections from Rh30 and RD tumors by immunostaining for CD31. Furin overexpression in Rh30 fur resulted in a slight increase in MVD compared to wild type Rh30 (Fig 3C; Rh30 wt: 40 vessels/mm^2^, Rh30 fur: 50 vessels/mm^2^). Conversely, reduced furin activity in Rh30 cells led to lower MVD (Fig 3C; Rh30 pdx: 25 vessels/mm^2^, Rh30 shFA: 30 vessels/mm^2^, and Rh30 shFE: and 20 vessels/mm^2^). Similar results were obtained for RD tumors (Fig 3F; RD wt: 38 vessels/mm^2^, RD fur: 61 vessels/mm^2^, RD pdx: 22 vessels/mm^2^). These results suggest a possible role of furin in the recruitment of new blood vessels at the early stage of RMS tumor growth, which, in turn, might account for the differences in tumor growth onset that were observed.

### Furin activity favors migration and invasion *in vitro*

**Fig 4:**
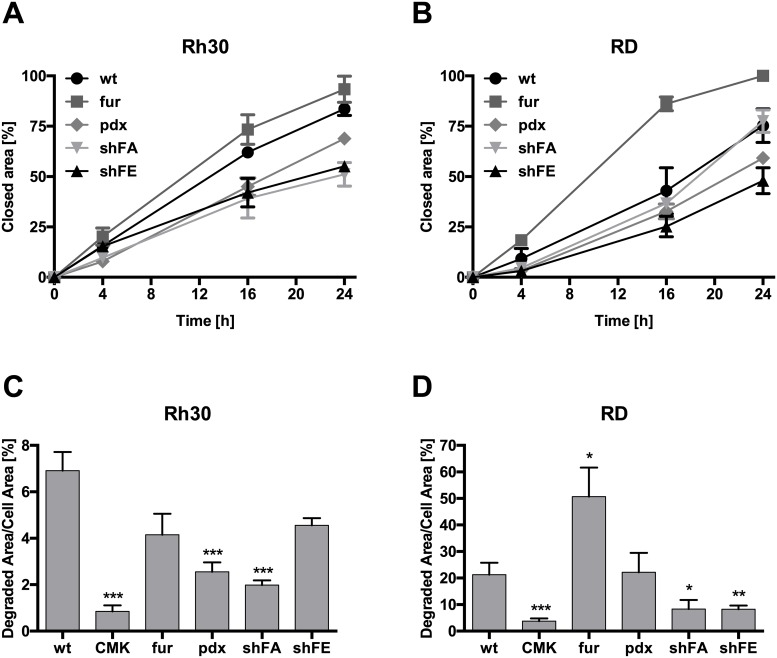
Migration and invasion of RMS cells is influenced by furin activity. A) Impact of furin activity on wound healing. Rh30 and RD cells with stable modulation of furin activity were used in a scratch assay. Pictures were taken at 0h, 4h, 16h and 24h and wound healing was assessed over time 0h using the program TScratch. Depicted are mean values ± SD. B) Effect of furin activity on RMS cell invasiveness. Rh30 and RD cells (wt, fur, pdx, shFA and shFE) were cultured on fluorescently labeled gelatin-coated 8-chamber slides for 24h and 48h, respectively. Treatment with 50 μM of the pan-PC inhibitor decanoyl-RVKR-chloromethylketone (CMK) was started 16h prior plating of the cells. Immunofluorescent pictures were taken and percentage of invasion was calculated based on degradation of fluorescent gelatin using the program ImageJ. Depicted are mean values ± SD. Student t-test, p<0.05 *, p<0.01 **, p<0.005 ***.

In order to further investigate the contribution of furin to the malignant phenotype of RMS cells, we studied migration and invasion abilities of Rh30 and RD cells with distinct furin activities *in vitro*. To investigate cell migration *in vitro*, we assessed wound closure over a timeframe of 24h. Rh30 wt cells migrated in general a little faster than RD wt cells (Fig 4A and 4B). In both cell lines, overexpression of furin led to an accelerated wound closure compared to the corresponding wild type cells. This effect was more pronounced in RD fur as compared to Rh30 fur. In contrast, inhibition of furin activity through expression of pdx or decreased expression by silencing of furin resulted in delayed wound closure for both Rh30 and RD cells. These results indicate a positive correlation between furin activity and the ability of RMS cells to migrate.

Next, we monitored the effect of furin on the ability of Rh30 and RD cells to invade a matrix. This was assessed with an invadopodia assay that allows assessment of cellular invasiveness by measuring the degradation of fluorescent gelatin. In addition to the previously described stable cell lines with modulated furin we also used the potent PC inhibitor decanoyl-RVKR-chloromethylketone (CMK) in this assay. In both RMS cell lines, Rh30 and RD, reduction of furin activity, either in stably modified cells (pdx or shRNA) or in wild type cells treated with CMK, resulted in diminished matrix degradation (Fig 4C and 4D). The gain in furin activity, on the other hand, led to increased degradation of the matrix in RD fur cells compared to RD wt. Surprisingly, Rh30 fur were less able to degrade gelatin than Rh30 wt, although not in a statistically relevant manner. This result is in line with previous *in vitro* and *in vivo* observations, where we did not observe an impact of furin overexpression in Rh30 on the cell phenotype, suggesting that in Rh30 cells furin activity appears to be already at a plateau and cannot be further increased. Taken together, these results point at a role of furin activity in migration and invasion processes in RMS cells.

### Furin regulates maturation of substrates important in RMS progression

**Fig 5:**
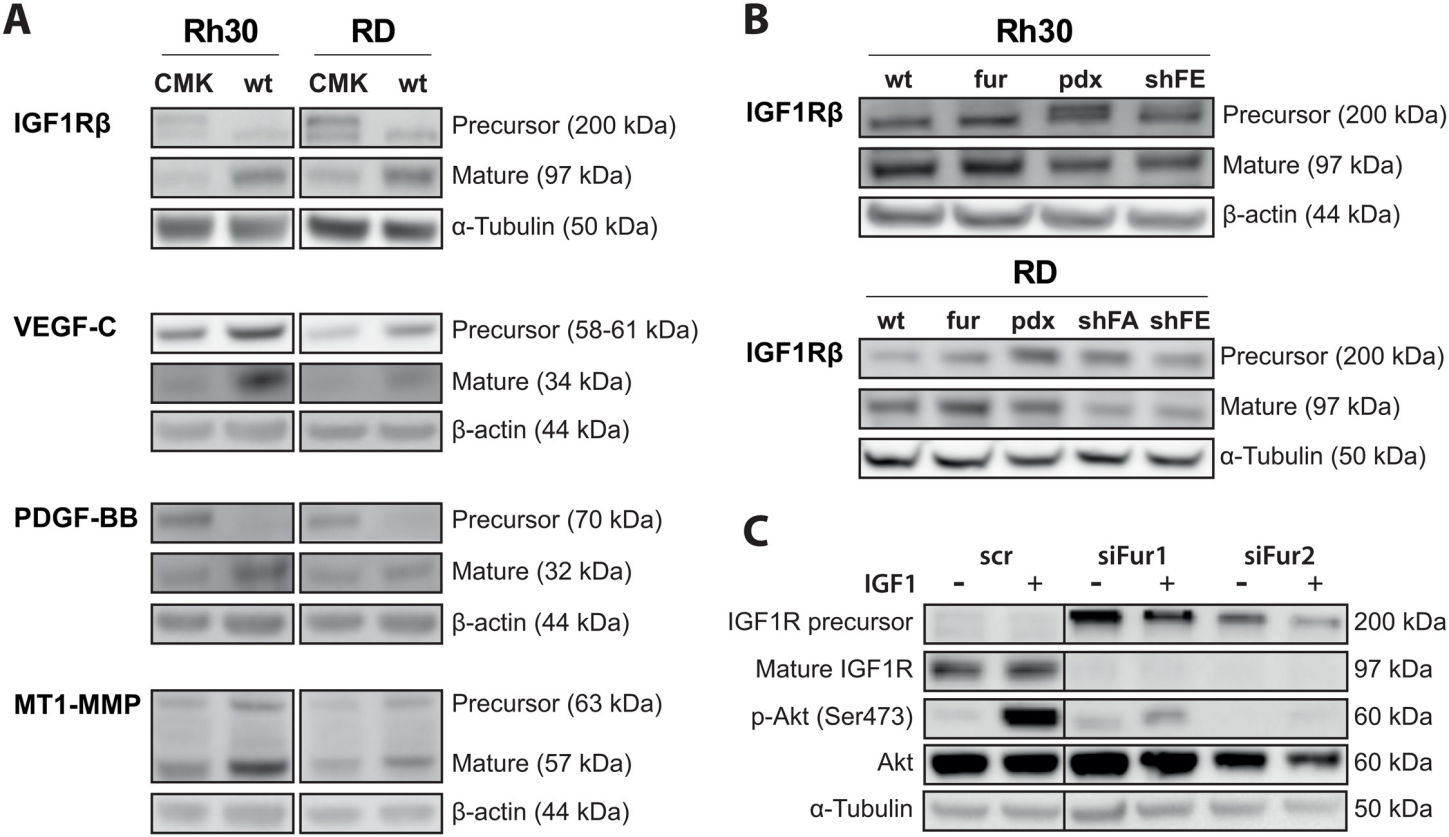
Maturation of furin substrates and IGF1R signaling are furin activity dependent in RMS cells. A) Maturation of furin substrates upon treatment with pan-PC inhibitor CMK. Rh30 and RD cells were treated with 50 μM CMK for 16h and maturation status of the furin substrates IGF1Rβ, VEGF-C, PDGF-BB and MT1-MMP were examined by immunoblotting. B) IGF1Rβ maturation in Rh30 and RD cell lines. Levels of precursor and mature IGF1Rβ were analyzed in stable Rh30 and RD cell lines (wt, fur, pdx, shFA and shFE) by immunoblotting. C) IGF signaling in RD cells upon transient furin silencing. RD cells were transiently transfected with control (scr) or anti-furin siRNA (siFur1 and siFur2) and serum starvation was started 24h post transfection. Cells were stimulated for 10 min with 50 ng/mL IGF1 48h post transfection and maturation status of IGF1Rβ and phosphorylation of Akt (Ser473) were assessed by immunoblotting.

To decipher the furin substrates involved in tumor growth, migration and invasion in RMS cells, we examined by immunoblotting the maturation status of IGF1Rβ, VEGF-C, PDGF-BB and MT1-MMP in Rh30 wt and RD wt cells upon inhibition of furin activity by the PC inhibitor CMK (Fig 5A). In the case of IGF1Rβ and PDGF-BB, we observed an accumulation of precursor and decrease in mature proteins upon inhibition of furin with CMK. For VEGF-C and MT1-MMP reduced furin activity resulted in lower levels of mature and precursor protein, suggesting enhanced degradation of unprocessed protein proforms. Thus, in RMS cells PCs seem to be involved in the maturation of all four investigated furin substrates. In addition to the use of the inhibitor CMK, we examined the maturation of IGF1Rβ in Rh30 and RD cells with stable modulation of furin activity by immunoblotting (Fig 5B). In both cell lines, we observed a slight increase in cleaved IGF1Rβ upon overexpression of furin. Conversely, a decrease of furin activity, either by expression of the inhibitor pdx or by silencing of furin, led to an accumulation of IGF1Rβ precursor and lower levels of mature IGF1Rβ (Fig 5B).

In order to examine the impact of furin activity on IGF signaling in RMS cells, and to exclude any type of effect arising during cell selection required for shRNA, we transfected RD cells with specific siRNA against furin (siFur1 and siFur2) and stimulated the cells with 50 ng/mL IGF1, 48h post transfection. As expected, silencing of furin severely impaired maturation of IGF1Rβ, leading to a decrease in mature form and accumulation of IGF1Rβ proform. Control siRNA transfected cells showed a high level of Akt phosphorylation upon stimulation with IGF1. In contrast, silencing of furin and subsequent loss of IGF1Rβ maturation mostly prevented stimulation of Akt phosphorylation, indicating an inability of RMS cells to trigger IGF signaling in the absence of furin activity (Fig 5C). Phosphorylation of Erk1/2 was generally not stimulation upon treatment with IGF1 (data not shown). Taken together our data suggest that furin activity is necessary to ensure processing of IGF1Rβ, VEGF-C, PDGF-BB and MT1-MMP and that maturation of IGF1Rβ by furin is required to maintain IGF signaling. Thus, furin has an important role in promoting the malignant phenotype of RMS cells by supporting the maturation and activation of proteins involved in, or enabling, vital cellular abilities such as proliferation or matrix degradation.

## Discussion

In this study, we show that inhibition of furin activity in rhabdomyosarcoma results in delayed tumor growth *in vivo*, and that loss of furin activity, through RNA interference or application of pan-PC inhibitors decreases migration and invasion *in vitro*. Furthermore, reduced furin activity results in diminished processing of furin substrates important for the oncogenic phenotype of rhabdomyosarcoma and in the abrogation of IGF signaling.

We observed delayed RMS tumor growth upon engraftment of mice with Rh30 and RD cells with reduced furin activity, achieved either by silencing or by expression of the pan-PC inhibitor α1-PDX/pdx [34]. We hypothesized that this could be the result of reduced growth factor signaling and/or impaired vessel formation. We could show that proteolytic activity of furin in RMS cells is required to process the potent mitogen PDGF-BB and the growth factor receptor IGF1R. We could also show that this results in decreased IGF signaling. Similarly, furin inhibition in carcinoma cells diminishes PDGF-A and IGF1R processing and abolishes stimulation of related signaling pathway [35]. IGF signaling pathway is particularly important in RMS. Increased IGF2 and IGF1R levels are key to a strong mitogenic signalling loop that promotes sustained proliferation as well as survival of RMS cells [36].

In order to grow beyond a certain size, a tumor has to initiate angiogenesis, a step in tumorigenesis that is known as angiogenic switch [37]. Furin activity has been shown to promote tumor vascularization [5,8,38]. Stimulation of vessel formation is dependent on secretion of pro-angiogenic growth factors like VEGF-C and VEGF-D, both of which require processing through furin or furin-like PCs to mature and stimulate VEGF signaling and angiogenesis or lymphangiogenesis [39,40]. Accordingly, maturation of VEGF-C was shown to be necessary for tumor formation [41]. Likewise, secretion of mature PDGF-BB favors tumor angiogenesis through paracrine stimulation of PDGFR-β on endothelial cells, leading to proliferation, migration and sprouting [42]. Hence, decreased VEGF-C and PDGF-BB processing, as confirmed *in vitro*, is likely responsible for reduced microvessel density *in vivo*, and delayed the angiogenic switch. We therefore suggest that the delay in initial tumor growth observed in RMS tumors with reduced furin activity may be attributed to impaired furin substrate processing.

Furthermore, we found that activity of furin is important for the migratory and invasive behavior of RMS cells *in vitro*. Hence, we observed reduced mobility in wound healing assays and reduced invasion in gelatin degradation. Interestingly, we observed also decreased processing of the furin substrate MT1-MMP, an important enzyme for breakdown of extracellular matrix and metastasis. Furin is also responsible for the activation of other matrix metalloproteases including ADAMs [43] and ADAM-TS [44] and indirectly mediates MMP2/gelatinase A activation, since active MT1-MMP is the main enzyme responsible for processing of pro-MMP2 on the cell surface [45]. Additionally, autocrine PDGF signaling was shown to promote the metastatic potential of breast cancer and pancreatic cancer cells [46,47]. Therefore, furin-mediated PDGF processing in RMS cells might further favor their metastatic behavior. Thus, several lines of evidence support the notion that PCs, and in particular furin, activate metastasis-associated proteins. For instance, furin-mediated catalytic activation of MT1-MMP has been linked to progression of head and neck squamous cell carcinomas [14,48]. Similar to our studies, others showed that inhibition of furin decreases cell motility, migration and invasiveness of breast cancer, lung adenocarcinoma, fibrosarcoma and osteosarcoma cells *in vitro* [49,50,51,52] and proposed furin inhibition for prevention of metastasis [17].

In conclusion, our study identifies a role of furin activity in RMS, which is mediated via proteolytic activation of key substrates involved in proliferation, motility and dissemination of RMS cells. We confirmed furin-dependent processing of IGF1R, PDGF-BB, VEGF-C and MT1-MMP in RMS cells; however, it is likely that other furin substrates are involved. Based on our findings and studies from other groups, we predict that furin inhibition may be of benefit for treatment of patients with RMS or other cancer types.

## Supporting Information

S1 Fig Furin mRNA levels in RMS cell lines. A) Endogenous furin mRNA levels were determined by qRT-PCR in 5 eRMS and 15 aRMS cell lines. Expression levels relative to GAPDH are shown.
(PDF)

S2 Fig Expression of furin in RMS cell lines. Original blot image from Fig 2B. Original image is in the left, contrast enhanced image is on the right. A) Rh30 cells. Loading left to right: Rh30 wt, fur, pdx, shFA, shFE. B) RD cells. Loading left to right: RD wt, fur, pdx, shFA, shFE.
(PDF)

S3 Fig Furin levels in Rh30 and RD xenografts. A) 5x10^6^ RMS cells with stable modulation of furin activity were s.c. injected in NOD/Scid IL2reg-/- mice and tumor growth was monitored over time. Tumors were excised upon a size of 750–1000 mm3 and furin mRNA levels were measured by RT-qPCR. Values shown are normalized over GAPDH. Rh30 wild type was set to 100%. Protein levels were assessed by immunoblot, shown are two independent immunoblots performed with different tumor extracts. B) Expression of furin in RMS tumor xenografts. Immunohistochemistry was performed on sections from tumors collected upon reaching a size of 750–1000 mm^3^ (endpoint).
(PDF)

S1 Table Proprotein convertase mRNA expression in pediatric sarcoma cell lines. (PDF)
