# Supplementary material for: The Proprotein Convertase Furin Contributes to Rhabdomyosarcoma Malignancy by Promoting Vascularization, Migration and Invasion
Source: PLoS One. 2016 Aug 22;11(8):e0161396. doi: 10.1371/journal.pone.0161396 (PMC4993484; doi:10.1371/journal.pone.0161396)
Supplement: S1 Fig — A) Endogenous furin mRNA levels were determined by qRT-PCR in 5 eRMS and 15 aRMS cell lines. Expression levels relative to GAPDH are shown. (PDF) [file pone.0161396.s001.pdf]

S1 Fig. Furin mRNA levels in RMS cell lines

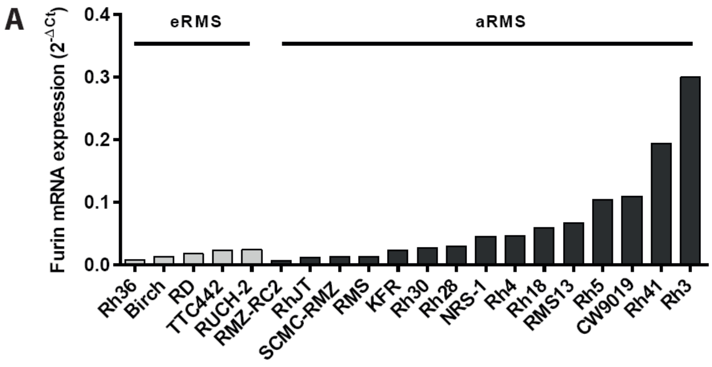

S1 Fig. Furin mRNA levels in RMS cell lines. A) Endogenous furin mRNA levels were determined by qRT-PCR in 5 eRMS and 15 aRMS cell lines. Expression levels relative to GAPDH are shown.
