# Supplementary material for: The Proprotein Convertase Furin Contributes to Rhabdomyosarcoma Malignancy by Promoting Vascularization, Migration and Invasion
Source: PLoS One. 2016 Aug 22;11(8):e0161396. doi: 10.1371/journal.pone.0161396 (PMC4993484; doi:10.1371/journal.pone.0161396)
Supplement: S2 Fig — Original blot image from Fig 2B. Original image is in the left, contrast enhanced image is on the right. A) Rh30 cells. Loading left to right: Rh30 wt, fur, pdx, shFA, shFE. B) RD cells. Loading left to right: RD wt, fur, pdx, shFA, shFE. (PDF) [file pone.0161396.s002.pdf]

S2 Fig. Expression of furin in RMS cell lines

A

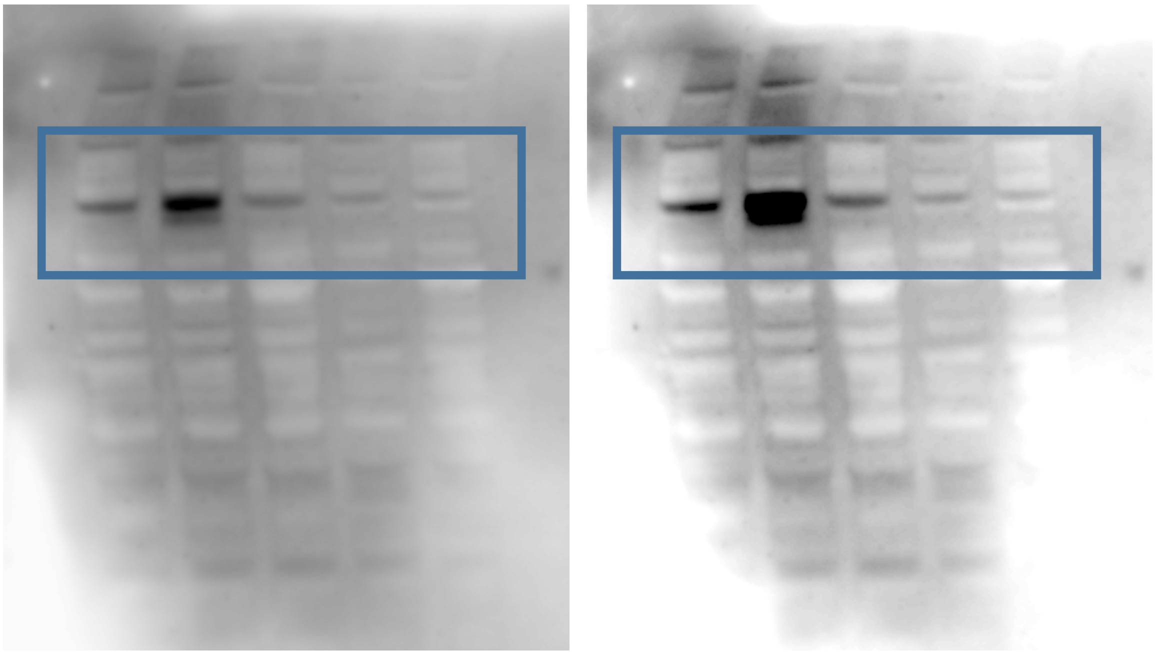

B

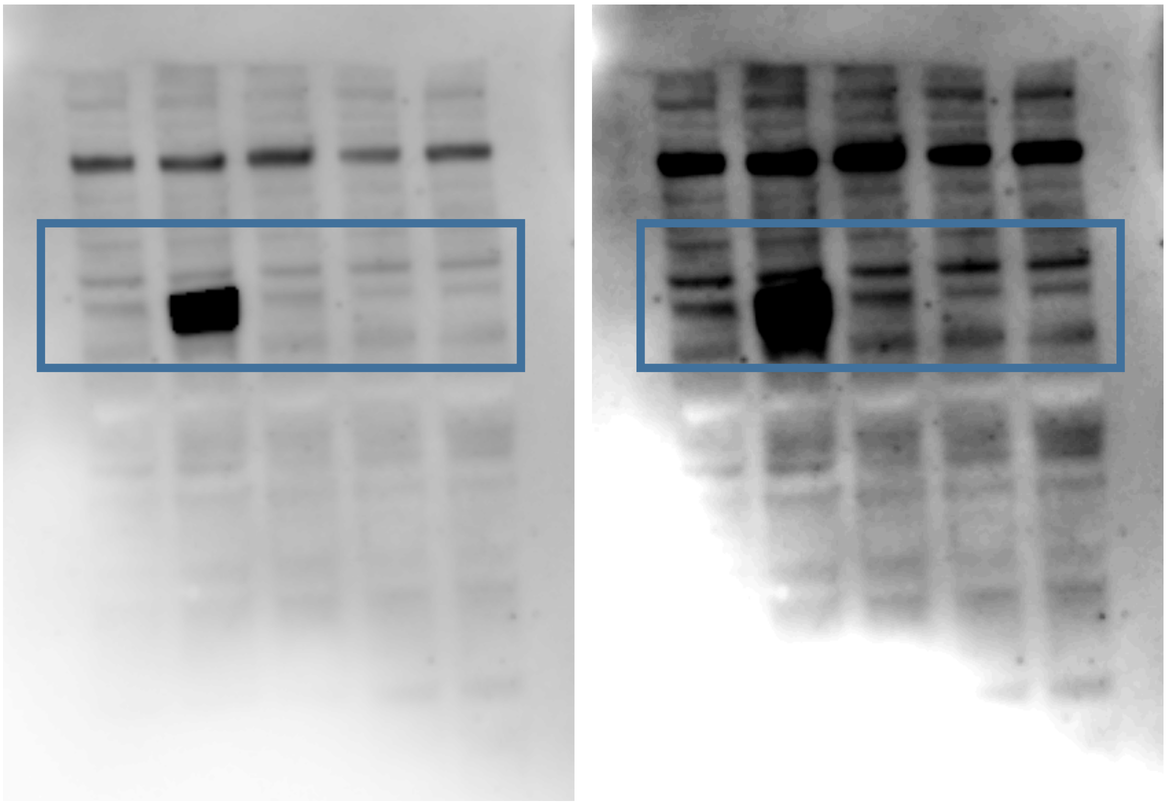

S2 Fig. Expression of furin in RMS cell lines. Original blot image from Figure 2B. Original image is in the left, contrast enhanced image is on the right. A) Rh30 cells. Loading left to right: Rh30 wt, fur, pdx, shFA, shFE. B) RD cells. Loading left to right: RD wt, fur, pdx, shFA, shFE.
