# Supplementary material for: The Proprotein Convertase Furin Contributes to Rhabdomyosarcoma Malignancy by Promoting Vascularization, Migration and Invasion
Source: PLoS One. 2016 Aug 22;11(8):e0161396. doi: 10.1371/journal.pone.0161396 (PMC4993484; doi:10.1371/journal.pone.0161396)
Supplement: S3 Fig — A) 5x10^6^ RMS cells with stable modulation of furin activity were s.c. injected in NOD/Scid IL2reg-/- mice and tumor growth was monitored over time. Tumors were excised upon a size of 750–1000 mm3 and furin mRNA levels were measured by RT-qPCR. Values shown are normalized over GAPDH. Rh30 wild type was set to 100%. Protein levels were assessed by immunoblot, shown are two independent immunoblots performed with different tumor extracts. B) Expression of furin in RMS tumor xenografts. Immunohistochemistry was performed on sections from tumors collected upon reaching a size of 750–1000 mm3 (endpoint). (PDF) [file pone.0161396.s003.pdf]

S3 Fig. Furin levels in Rh30 and RD xenografts.

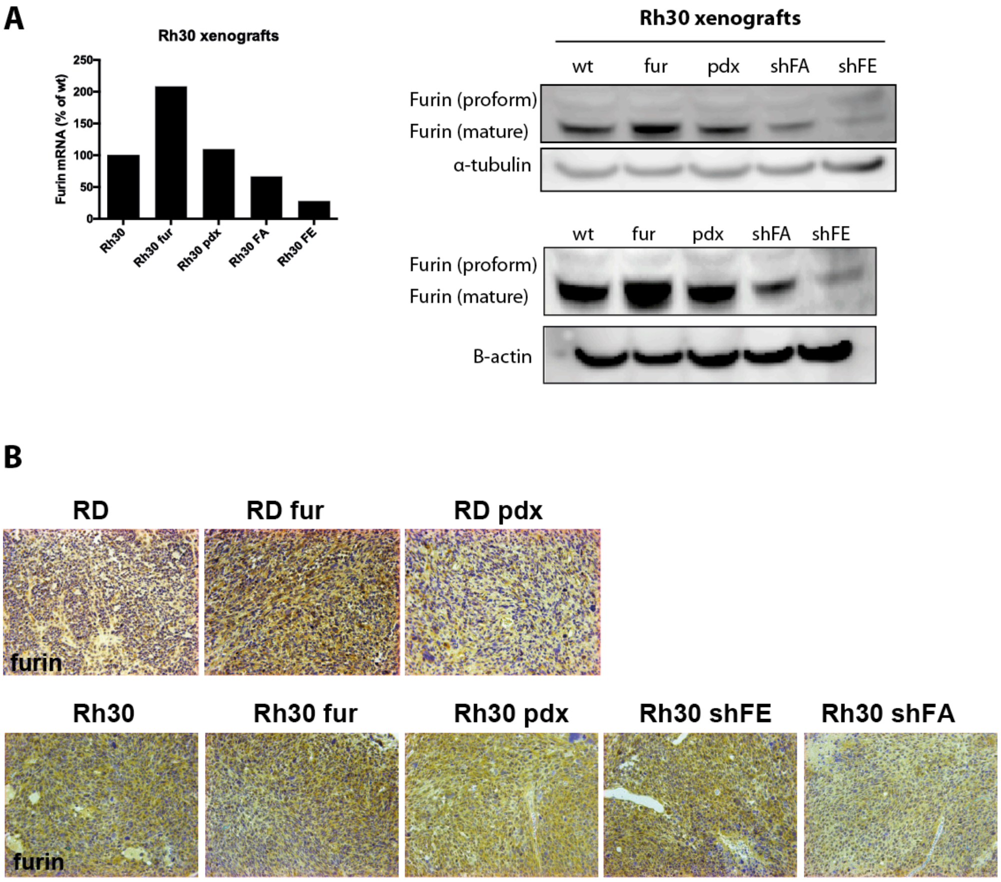

S3 Fig. Furin levels in Rh30 and RD xenografts. A) $5x10^6$ RMS cells with stable modulation of furin activity were s.c. injected in NOD/Scid $IL2reg^{-/-}$ mice and tumor growth was monitored over time. Tumors were excised upon a size of 750-1000 mm^3 and furin mRNA levels were measured by RT-qPCR. Values shown are normalized over GAPDH. Rh30 wild type was set to 100%. Protein levels were assessed by immunoblot, shown are two independent immunoblots performed with different tumor extracts. B) Expression of furin in RMS tumor xenografts. Immunohistochemistry was performed on sections from tumors collected upon reaching a size of 750-1000 mm^3.
