# Supplementary material for: The Proprotein Convertase Furin Contributes to Rhabdomyosarcoma Malignancy by Promoting Vascularization, Migration and Invasion
Source: PLoS One. 2016 Aug 22;11(8):e0161396. doi: 10.1371/journal.pone.0161396 (PMC4993484; doi:10.1371/journal.pone.0161396)
Supplement: S1 Table — (PDF) [file pone.0161396.s004.pdf]

S1 Table. Proprotein convertase mRNA expression in pediatric sarcoma cell lines.

Cell line	Proprotein convertase								
	PC1/3	PC2	Furin	PC4	PC5	PACE4	PC7	S1P	PCSK9
**Ewing sarcoma**									
TC71	-11.57	-5.01	-5.88	-9.70	-16.61	-8.29	-8.43	-6.51	-6.16
RD ES	nd	-5.92	-6.06	-10.57	-16.61	-9.29	-8.08	-5.57	-8.59
SK ES	nd	-6.75	-6.78	-12.61	-14.02	nd	-9.12	-7.73	-6.72
SKMNC	nd	-8.08	-5.80	-12.22	-9.98	-8.20	-8.97	-7.12	-9.83
A673	-11.29	-4.97	-5.80	-11.48	-15.02	-11.36	-8.59	-6.51	-6.51
**Osteosarcoma**									
SAOS	-12.44	nd	-4.68	-13.80	-11.57	nd	-7.06	-5.57	nd
LM5	-15.61	nd	-4.68	-15.61	-12.36	nd	-7.10	-5.80	nd
HOS	nd	nd	-4.64	-11.09	-11.15	-8.64	-6.38	-4.76	nd
143B	nd	nd	-5.44	-12.36	-10.25	-8.43	-8.43	-6.06	nd
MG63	-15.61	nd	-6.27	-13.29	-12.22	-9.29	-9.38	-6.72	nd
M8	-15.61	nd	-6.64	-12.09	-11.15	-8.90	-8.97	-6.64	nd
HU09	-11.66	nd	-4.92	-14.02	-8.43	-12.70	-7.86	-6.06	nd
M132	-9.70	nd	-4.16	-12.09	-7.36	-16.61	-7.51	-5.64	-16.61
**Embryonal rhabdomyosarcoma**									
RD	-5.03	nd	-5.80	-12.36	-8.08	nd	-8.59	-6.16	-15.61
Rh36	nd	-8.53	-6.97	-11.06	-9.83	-9.29	-9.04	-6.83	-16.61
TTC442	-16.61	nd	-5.44	-12.44	-12.91	-15.61	-7.14	-6.51	-11.57
Birch	-14.61	-6.93	-6.27	-10.27	-12.29	-8.64	-8.76	-6.38	nd
RUCH-2	-7.20	nd	-5.38	-12.70	-12.02	nd	-7.76	-5.57	-16.61
**Alveolar rhabdomyosarcoma**									
Rh18	nd	-7.70	-4.06	-11.25	-13.80	-9.20	-9.97	-4.24	nd
Rh30	nd	nd	-5.21	-13.15	-8.90	nd	-7.83	-6.72	nd
Rh3	-16.61	-5.88	-1.74	-13.29	-14.29	-2.64	-7.97	-3.32	-7.24
Rh4	nd	-8.83	-4.41	-13.15	nd	-5.64	-8.83	-4.06	-15.02
Rh5	-16.61	-10.54	-3.32	-11.97	-12.09	-4.88	-8.16	-3.18	-16.61
Rh41	nd	nd	-2.40	-12.80	-10.48	-5.16	-7.93	-2.32	-12.91
RMS13	nd	nd	-5.21	-15.61	-8.83	nd	-9.70	-4.32	-13.15
CW9019	nd	nd	-3.18	nd	nd	nd	-8.29	-4.76	nd
SCMC-RMZ	nd	nd	-6.27	-11.80	-12.15	-4.97	-7.00	-5.38	-5.06
KFR	nd	nd	-5.44	-8.70	-10.59	-3.18	-8.04	-4.18	-12.70
RhJT	nd	nd	-6.38	-11.40	nd	-4.54	-7.64	-5.32	-12.29
RMS	nd	-9.59	-6.27	-12.70	nd	-4.08	-8.33	-5.44	nd
RMZ-RC2	nd	-11.22	-7.33	nd	nd	-4.24	-8.16	-5.38	-10.12
NRS-1	nd	-8.16	-4.47	nd	-12.02	-2.74	-6.97	-5.97	-7.56
Rh28	nd	-3.78	-5.06	-11.66	nd	-1.43	-7.38	-5.38	-3.66

Data were normalized to GAPDH expression. Depicted are -ΔCt values. nd= no expression detected.
